# Investigations on the Fatigue Behaviour of 3D-Printed Continuous Carbon Fibre-Reinforced Polymer Tension Straps

**DOI:** 10.3390/polym14204258

**Published:** 2022-10-11

**Authors:** Tadej Vidrih, Peter Winiger, Zafiris Triantafyllidis, Valentin Ott, Giovanni P. Terrasi

**Affiliations:** 1 Mechanical Systems Engineering Laboratory, Swiss Federal Laboratories for Materials Science and Technology, Überlandstrasse 129, 8600 Dübendorf, Switzerland; 2 Structural Engineering Laboratory, Swiss Federal Laboratories for Materials Science and Technology, Überlandstrasse 129, 8600 Dübendorf, Switzerland

**Keywords:** 3D-printed continuous carbon fibre-reinforced polymer composites, CFRP, fused filament fabrication, polyamide PA12, pin-loaded strap, tensile fatigue, fretting fatigue

## Abstract

The focus of this research is an investigation on the fatigue behaviour of unidirectional 3D-printed continuous carbon fibre-reinforced polymer (CFRP) tension straps with a polyamide matrix (PA12). Conventionally produced tension straps are becoming established components in the mechanical as well as the civil engineering sector, e.g., as rigging systems for sailing boats and cranes and—recently introduced—as network arch bridge hangers. All these structures are subjected to high fatigue loads, and although it is commonly reported that carbon fibre-reinforced polymers show excellent fatigue resistance, there is limited understanding of the behaviour of CFRP loop elements under such loads, especially in combination with fretting at the attachment points. Research on this topic was performed at Empa in the past decade on thermoset CFRP straps, but never before with 3D-printed continuous CFRP straps with a thermoplastic matrix. This paper examines an additive manufacturing and post-consolidation method for producing the straps and presents initial results on their fatigue performance, which show that the fatigue endurance limit of the investigated 3D-printed and post-consolidated CFRP strap design is acceptable, when compared to steel tendons. However, it is still 20% lower than conventionally produced CFRP straps using out-of-autoclave unidirectional carbon fibre prepregs. The reasons for these findings and potential future improvements are discussed.

## 1. Introduction

**Figure 1:**
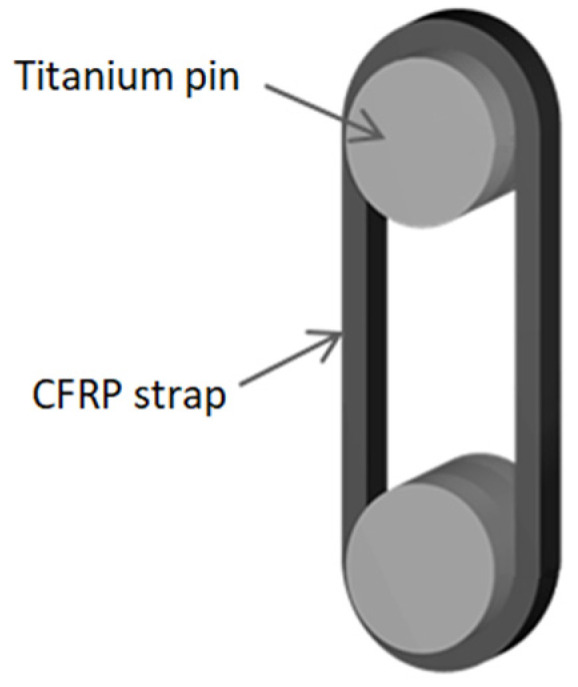
Schematic representation of a pin-loaded strap.

Carbon fibre-reinforced polymers (CFRP) are a strong competitor to the conventional steels used for tensile structural elements and are becoming increasingly attractive in the construction industry due to their outstanding mechanical performance, lower weight, durability, and sustainability [1]. CFRP has the potential to replace steel ropes and cables, as they are prone to corrosion from environmental exposure that compromises their overall performance and causes substantial expenditure for corrosion protection measures and periodical renewal [2]. Several structures that implement CFRP tensile elements have been constructed so far, a review of which is presented by Liu et al. [3]. A recent, world-first example of a large bridge (127 m span) that fully relies on CFRP hangers is the network arch light rail bridge installed in May 2020 over the A8 motorway in Stuttgart, Germany [4], the deck of which is entirely supported by 72 pin-loaded, unidirectional CFRP strap elements. Another rail bridge with a similar configuration (network arch with CFRP strap hangers, 130 m span) is currently under construction at the Küstrin-Kietz rail crossing over the Oder river at the German–Polish border and is expected to be completed in 2023 [5]. Due to the low weight of the CFRP hangers, no cranes or supporting pillars were required in the installation process, since they can be easily lifted by hand and installed by two workers from a mobile lifting platform. Moreover, the construction was both more economical and more sustainable than an analogous network arch bridge with flat steel hangers [4]. This project was groundbreaking in terms of using CFRP as the sole material for the tensile elements supporting the bridge deck, particularly since hangers in such bridges are subjected to high tensile fatigue loads. This motivated the Swiss Federal Laboratories for Material Science and Technology (Empa) previously to investigate the fatigue behaviour of pin-loaded CFRP straps, with a specific focus on the fretting phenomena that are present in this application due to the constant relative motion between the loading pin’s surface and the curved portion of the CFRP strap at the connection points (Figure 1). Previous experimental studies concerned strap specimens that were laminated using the same materials and a scaled-down geometry from those used in the above-mentioned network arch bridge [6]. These focused on the fatigue performance of the CFRP straps that were fretted against titanium and CFRP pins at room temperature [7] and elevated service temperatures [8], as well as on their thermomechanical behaviour at high temperatures that are representative of accidental load cases (i.e., fire) [9]. In the study presented herein, a new method of fabrication of CFRP straps based on 3D printing is explored in order to investigate its potential and durability limits against the conventional tape laying and out-of-autoclave lamination techniques that are currently the norm in composite manufacturing. In particular, the fretting fatigue behaviour of the novel straps anchored with titanium pins was studied with a comprehensive series of tensile fatigue experiments on small-scale specimens performed at 23 °C in air. An elastic stress analysis after Schürmann [10] was confirmed to be an appropriate design tool to capture the triaxial stress state in the vertex area at the onset of the strap’s curvature for the pin-loaded element.

### 1.1. 3D-Printing of Continuous Fibre-Reinforced Polymer Composites

Several types of CFRP straps are possible, which can be conventionally produced with either lamination, pultrusion, tape-winding, or pull-winding [3]. In this project, the tension straps were manufactured by 3D-printed continuous CFRP filaments that were subsequently stretched and compacted in a mould before being tested for quasi static tensile strength and under tensile fatigue loading. Three-dimensional printing of continuous CFRP is a relatively new approach to composite manufacturing and has been a topic in research and development in the past ten years. However, based on a recent literature review published by Sanei and Popescu [11], this technique needs to be thoroughly investigated, especially with respect to fatigue resistance. By implementing a 3D printing process for CFRP, one increases the production flexibility by continuously changing the direction of the fibres through the part with a precise and repeatable fibre deposition without needing a complex mould to produce a preform. There are many different approaches to the 3D printing of CFRP parts with a thermoplastic polymer matrix; these can be categorised by the type of reinforcing fibres (chopped fibres versus continuous tows) and the process of introducing the polymer matrix in the printed composite material (e.g., fusion of pre-impregnated carbon fibre filaments, versus impregnation of dry tows within the extrusion head or on the printing platform itself) [12,13,14]. After the manufacturing of the part by 3D printing, post-processing of the preform is required in the 3D printing methods developed by the composites industry in the last decade [14,15,16,17]. In particular, it is crucial to perform compression of the printed preform at an elevated temperature to achieve the final geometry and to increase the fibre volume fraction while minimizing porosity and therewith increasing the mechanical properties of the part to a level acceptable for a structural application [18]. In the opinion of the authors, this additional and necessary post-processing step is a serious limitation of today’s 3D printing FRP composite technologies because it adds considerable cost and component production time.

### 1.2. Fatigue Behaviour of CFRP Straps

Fatigue crack propagation and damage modes of CFRP are considerably different and more complex than those in isotropic materials, due to factors such as the anisotropic behaviour of the carbon fibres, the viscoelastic nature of the matrix, the fibre–matrix interaction, the layup sequence, and stress concentrations at the load introduction (anchorage) areas. Regardless of the numerous factors influencing the fatigue behaviour, Reifsnider [19] distinguished three different stages in the fatigue damage of multiaxial fibre-reinforced composites (FRPs). In the first stage, the laminate experiences severe matrix cracking in the off-axis fibre orientation, generally within the first 10–15% of the laminate’s life. At the end of the first stage, intralaminar matrix cracking reaches a uniform saturation spacing. The second stage incorporates up to 80% of the fatigue life, and the damage progression continues, however, at a much slower rate. Stage three is reached when the damage propagation increases for the second time and ends with the failure of the composite. There has been scant research in recent years on the tensile fatigue behaviour of 3D-printed continuous fibre-reinforced thermoplastic composites [20,21]. In [20], the upper stress levels of continuous carbon, glass, and aramid fibre-reinforced nylon were studied in the time domain of the SN curve and reached a maximum of a rather modest 130 MPa for a unidirectional (UD, i.e., 0°) carbon fibre arrangement loaded in the fibre direction failing at 80,000 load cycles. The loading stress ratio was set at R = 0.1 in tensile–tensile load condition. On the contrary, promising initial results were presented in [21] on the tensile fatigue behaviour (at R = 0 and with a loading frequency of 2 Hz), for flat UD carbon fibre-reinforced polyamide strips printed with a device from [17]. The obtained SN curve (with a Ps = 50% probability of survival) showed relatively high maximum stress values of 718 MPa achieved for a fatigue life of 293,000 load cycles. Serious limitations in this work were the very low amount of tested fatigue specimens (only five strips at different load levels) and the very low specimen cross-section area (3.9 mm^2^) and thickness of 0.6 mm.

Although the fatigue behaviour of FRP composites is complex on its own, in the case of pin-loaded straps it becomes even more complex due to the presence of fretting problems at the pin-to-strap interfaces. Fretting fatigue is a result of wear due to frictional contact between two components that are subjected to cyclic displacement relative to each other [22]. Friedrich et al. [23,24,25] conducted pioneering investigations on the fretting wear phenomena and fatigue life of carbon fibre-reinforced epoxy laminates, studying the different damage mechanisms for different fretting materials and laminate orientations. Cirino et al. [26] later also showed that fibre orientation and sliding direction have a strong influence on the abrasive wear behaviour of polymer composite materials and that the optimum wear resistance occurs when the sliding direction is normal to the fibre orientation, whereas material removal is greater when the fibres are oriented in the plane of the sliding surface.

The recent Empa studies of Baschnagel et al. [6,7] investigated the fatigue performance of thermoset CFRP straps, in which the curved parts of the UD laminates were fretting under tensile fatigue loading against the anchoring pins. Scaled-down specimen models were used in these studies and were compared against three full-scale strap specimens identical to the actual bridge hangers described in [4]; the fatigue tests were performed at a frequency of 10 Hz and stress ratio R of 0.1. Microscopic investigations of the small-and full-scale strap specimens revealed carbon fibre thinning and fibre–matrix debris agglomerating in the vertex area of the straps after failure [7]. The observed fretting products on the pins and straps included mostly short broken fibres, and carbon and resin particles that were attached to their surfaces. The reported ultimate failure mode was delamination that initiated at the end of the straps’ overlap and progressed towards the curved (pin) area, followed by fibre fracture. Overall, the fatigue behaviour of the straps was excellent, the endurance limit being at 750 MPa for straps sustaining a minimum of 3 × 10^6^ load cycles. This allowed the team led by Meier [4] to be granted the “structural design type approval” by the relevant German authorities, which was necessary for the construction of the world-first bridge fully relying on CFRP strap hangers in over the A8 motorway Stuttgart in 2020.

### 1.3. Aim and Scope

This research study investigates the efficiency of 3D printing fabrication of continuous, unidirectional CFRP straps with thermoplastic matrix using a Fused Filament Fabrication (FFF) technology [16] and followed by a post-printing compaction process, when compared with the conventional strap fabrication process using out-of-autoclave thermoset prepregs. It seeks to answer whether the purported precision and production efficiency in fabricating composite parts using this robotic fabrication technology offers improved mechanical performance for the case of pin-loaded CFRP straps.

This was addressed by setting up a small-scale production process for a feasibility study using a bench-top 3D printer setup [16] and by developing a post-printing compaction process (i.e., a stretching jig and compaction mould) at elevated temperature. To investigate the effects of 3D printing with respect to mechanical behaviour, the quasistatic and tensile fatigue performance of the 3D-printed straps was examined. This was done on strap specimens with the same geometric proportions as for the conventional thermoset-matrix straps investigated previously in [6,7] (and by following the same scaling down principles from the full-scale bridge straps in [4]).

## 2. Materials and Methods

**Figure 2:**
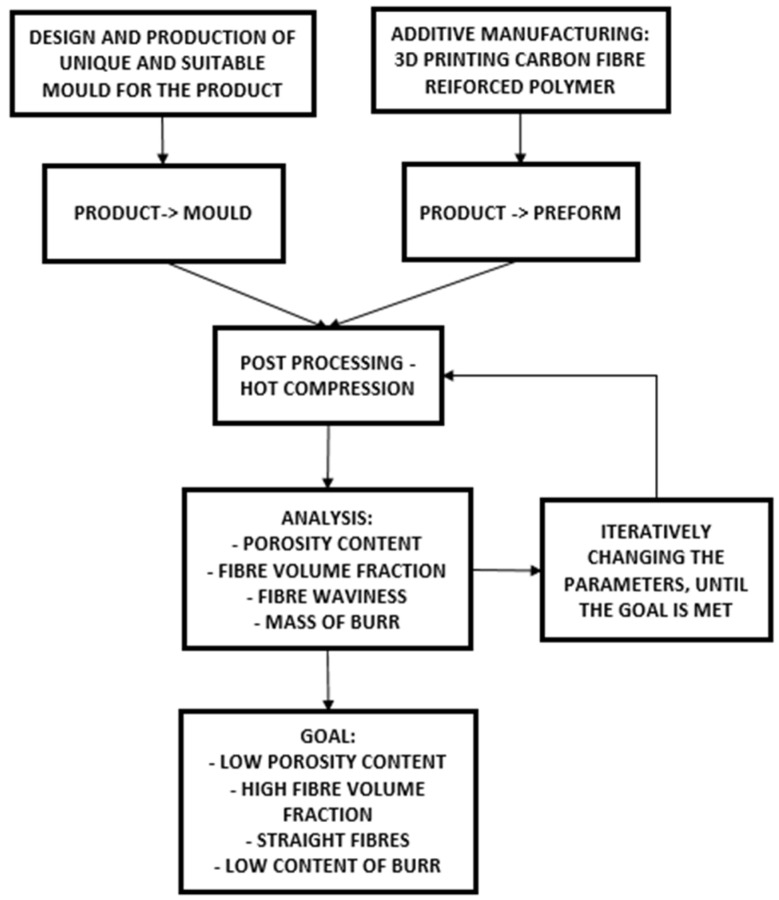
Iterative procedure for determining optimal post-processing parameters.

In order to fabricate a continuous 3D-printed CFRP tension strap, a reliable post-fusion process (post processing) needed to be established, which is fundamental for improving the mechanical properties of the preform and fully exploiting the material [15]. The process for determining the optimal post-processing window (Figure 2) can be divided into three steps:

1. Printing the specimens, where a preform is manufactured via FFF.
2. Transverse compression of the preform in a suitable mould at elevated temperature (around the melting temperature of the thermoplastic matrix) in order to achieve the final shape of the part, reduce fibre waviness of the looped strap and decrease its void content. Additional analysis with the optical microscope is necessary in order to verify the compaction results.
3. Iteratively changing the parameters of the post-processing step 2, until a low porosity content of the part with a reasonably low mass of the burr is achieved.

### 2.1. Manufacturing Process and Materials Used

To gain an understanding of the optimal post-processing parameters, small square unidirectional (UD) CFRP specimens of dimensions 60 × 20 × 5 mm were fabricated. They were printed on an Ultimaker2+ 3D printer with an additional CarbonKit unit (9T Labs, Zurich, Switzerland). These devices were purchased from 9T Labs, a spin-off company of ETH Zürich [15,16]. The 3D printing method used in this study is based on a fused filament fabrication principle, with some additional changes to the printer, such as a modified extrusion head with cutting mechanism, improved puller wheels, and a proprietary 9T Labs control software.

**Figure 3:**
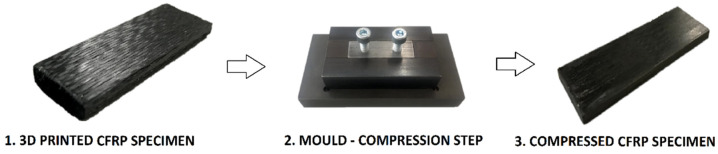
Manufacturing steps of CFRP specimens used to optimize post processing.

The material used was pre-impregnated CFRP filament with 60% fibre volume fraction of AS4 carbon fibre [27] and a PA12 matrix [28], also delivered by 9T Labs (Zurich, Switzerland). The three manufacturing steps for producing small square UD CFRP specimens are depicted in Figure 3.

### 2.2. Post-Processing Parameters

Post processing consists of compressing the part in a suitable mould at an elevated temperature. In order to determine the optimal processing window, a constant temperature of 210 °C (being 32 °C higher than the PA12 matrix melting temperature) and a dwell time of 10 min were set after getting advice from 9T Labs [15]. The effective pressure on the part was then gradually varied for different specimens. Based on the compression rate, mass of burr, and porosity analysis, the pressure was either decreased or increased, until a low porosity value of the specimen (<1%) with a reasonable low mass of burr (<6%) was reached.

In order to define the compression rate and the mass of burr, height, mass, and density measurements were required before and after the compression step. Density measurements were carried out following the EN ISO 1183-1 standard [29]. The compression rate δ can be calculated based on the following equation (Equation (1)), where $h_{i}$ represents the initial height and $h_{f}$ is the final height of the CFRP specimen.

(1) $$\delta =\left(1-\frac{h_{f}}{h_{i}}\right)\cdot 100\;\left[\%\right]$$

The relative mass of burr value $M_{b}$ can be similarly determined with values of $m_{i}$ as initial mass and $m_{f}$ as final mass of the specimen (Equation (2)).

(2) $$M_{b}=\left(1-\frac{m_{f}}{m_{i}}\right)\cdot 100\;\left[\%\right]$$

**Figure 4:**
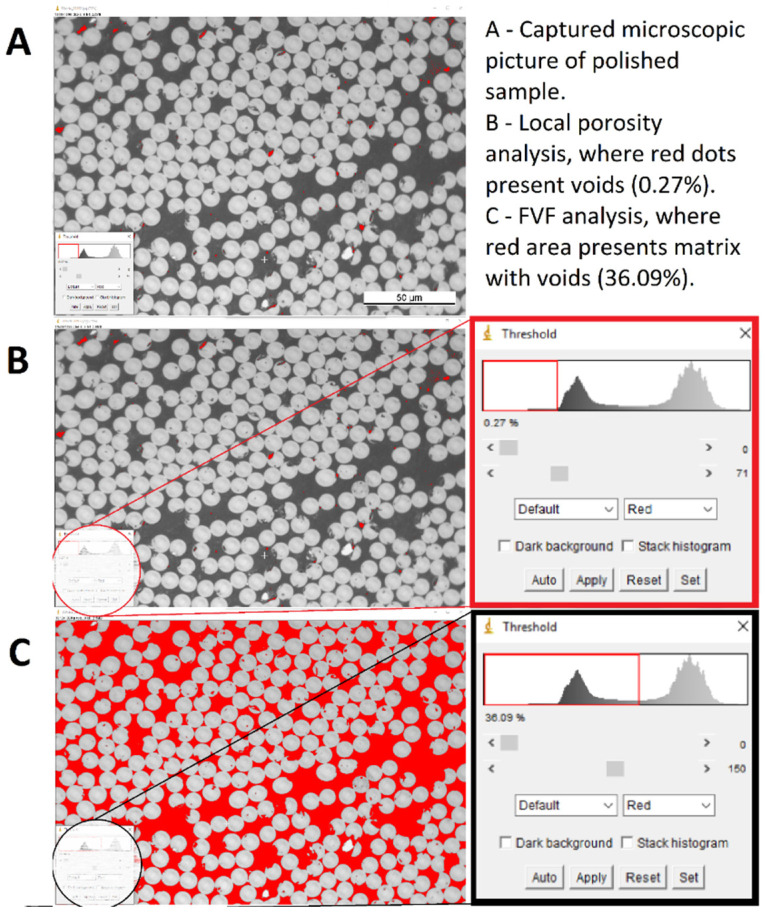
An example of determining local porosity and fibre volume fraction via optical microscopy. Light grey cross sections are AS4 carbon fibres and dark grey area is the PA12 matrix. Red spots in (**A**,**B**) are voids, while the red area in (**C**) represents the voids plus the PA12 matrix area.

The void and fibre volume contents were determined by local analysis of micro and macroscopic images. Micrographs were taken with an optical microscope ZEISS Axioplan (Zeiss Jena, Germany) and analysed with the ImageJ software [30] (Figure 4). Five specimens with different compression rates were tested at temperature 210 °C, and later two more at a decreased temperature of 200 °C.

### 2.3. Material Characterisation

#### 2.3.1. Manufacturing and Compression of Test Specimens

**Figure 5:**
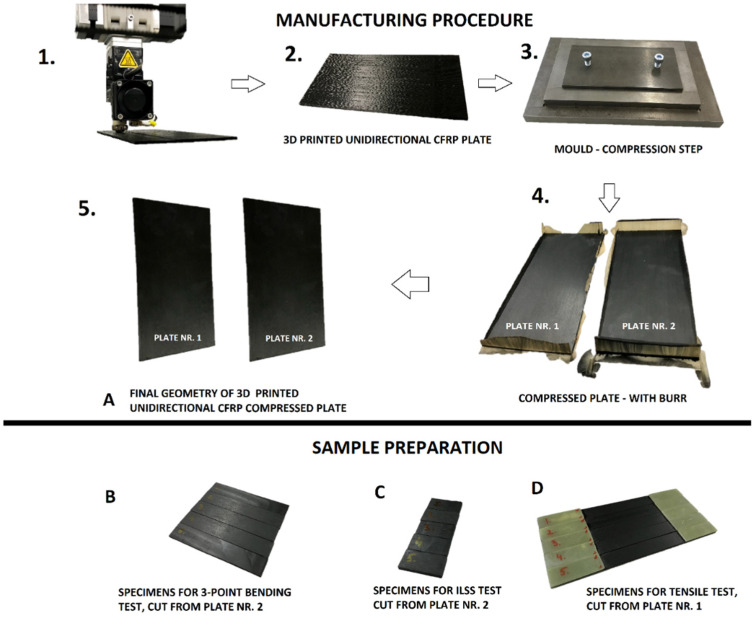
Manufacturing procedure (five steps) for material sample preparation. (**A**) is showing the final geometry of the 3D printed and compressed unidirectional CFRP plates. (**B**) depicts the cut specimens for the 3-point bending tests, (**C**) the specimens for the ILSS tests and (**D**) the tabbed specimens for the tensile tests in fibre direction.

Sample preparation was identical as in the previous step (see Figure 5, steps 1–5); however, instead of printing and post processing each individual specimen, larger plates were manufactured and later cut into an appropriate sample size according to the standards EN ISO 527-4 [31], EN ISO 14125 [32], and EN ISO 14130 [33] (see Figure 5A–D).

#### 2.3.2. Experimental Setup for Material Characterisation

After having determined the optimal compaction parameters and having obtained a stable printing process with reliable post processing, characterization of the printed unidirectional CFRP material followed. Tensile, flexural, and interlaminar shear strength (ILSS) tests were performed according to the standards EN ISO 527-4 [31], EN ISO 14125 [32], and EN ISO 14130 [33], respectively. An electrodynamic tensile testing machine (type Z010, Zwick GmbH, Ulm, Germany) was used to perform the ILSS and 3-point bending tests, while a servo-hydraulic test machine (type 1251, Instron^®^, Norwood, MA, USA) was used to perform the tensile tests. Some samples were additionally analysed for porosity under the optical microscope described in Section 2.2.

#### 2.3.3. Manufacturing of CFRP Tension Straps

**Figure 6:**
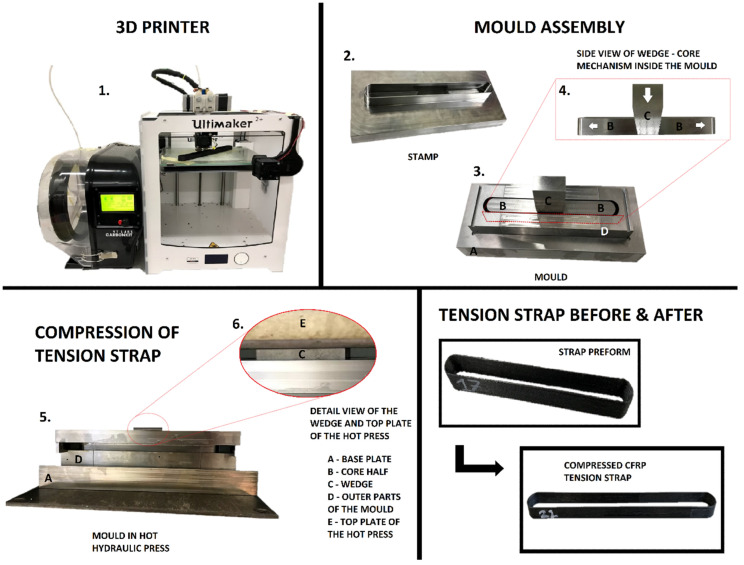
Manufacturing steps of 3D-printed CFRP straps with detailed mould assembly.

**Figure 7:**
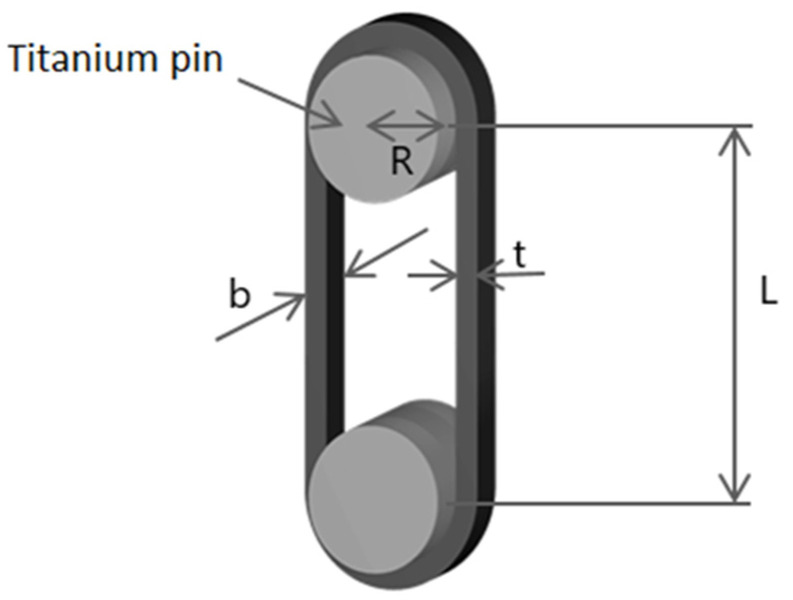
Strap dimensions.

After having developed a stable and reliable printing process and post-processing step (including however only transverse compaction), manufacturing and testing of the straps were developed (see Figure 6). Opposed to previous samples, tension strap specimens were 3D printed with one continuous (looped) motion, and no cutting of the fibres was necessary. Since the design of the strap involves two filament turns of 180°, some waviness of the fibres was present in the preform at its curvatures. In order to maximize the mechanical performance of the tension straps, longitudinal stretching of the fibres was therefore required. This was achieved in a further development of the post-processing step with a specially designed mould (see Figure 6) that was able to apply tensional forces from inside the strap to straighten up the fibres. The 3D-printed preform was placed into the mould cavity and then heated up in a hydraulic press. Because the mould includes an independently driven wedge and stamp, it was possible to apply the force separately to both elements. The wedge drove the two mould halves horizontally and therefore stretched the fibres (see Figure 6, numbers 4 and 6) to reach the final strap length of 220 mm (pin-to-pin length, Figure 7). When the level of the wedge reached alignment with the stamp, additional pressure was applied vertically and compression of the specimen was performed. This rather elaborate principle allowed unidirectional reinforced tension straps manufactured from one continuous CFRP strand to be obtained, with straight fibres and low void content. Due to the different geometry of the strap specimen and the different clearance between the stamp and the mould, the parameters of the post processing changed slightly compared to the flat coupon specimens. A fundamental difference was that the strap was compressed through its width while the coupon specimens were compressed through their thinner dimension, the thickness.

Finally, the 3D-printed and post-compacted CFRP straps specimens had the following dimensions (refer to Figure 7 for symbols): length L = 220 mm (±0.2 mm), thickness t = 1.3 mm (SD = 0.05 mm), and width b = 11.1 mm (SD = 0.4 mm).

#### 2.3.4. Experimental Procedure for the 3D-Printed and Post-Compacted CFRP Straps

The quasistatic tensile tests were performed on five strap preforms and six compressed tension straps following [31]. Tests were conducted on a servo-hydraulic test machine (type 1251, Instron^®^, Norwood, MA, USA). Tensile tests were carried out with cross-head speed of 2 mm/min. Fibre parallel strains were measured on a compressed tension strap with a linear encoder with a measuring length of 50 mm. Strain measurements, however, were not possible on the 3D-printed uncompressed preforms, as their tensile capacity was quite low and their scatter rather high, meaning that unpredictable failure could damage the strain gauge.

Additionally, a series of fatigue tests was performed on compressed straps at a frequency of 10 Hz and R = 0.1 for 28 additional tension straps and various loads using a servo-hydraulic test machine (type 1251, Instron^®^, Norwood, MA, USA). Specimens were exposed to upper tensile stress levels between 500 and 900 MPa until failure. The specimens that did not fail in fatigue after 3 million to 9 million load cycles were tested for their residual tensile strength following the above testing procedure (see Appendix A).

## 3. Results

### 3.1. Post-Processing Parameter Determination

Five specimens per pressure level were compressed at a temperature of 210 °C and under various compaction pressures (from 2 to 10 MPa). The average results are shown in the table below (Table 1). Corresponding standard deviations are given in Appendix D. The values of the compression ratio are between 41 and 29 % and decreased as the applied pressure was reduced. The fibre volume fraction (FVF) of the preform is 43.41%, and was increased to over 60% after post-processing. Even though the FVF of the used filament for printing is 60%, it was not possible to achieve the same value of FVF in the preforms, due to the high porosity content that is introduced during the 3D printing process. Post-processing decreases the void content and simultaneously increases the FVF. Similar results are reported when it comes to the mass of burr values because the amount of lost material decreases with reduced pressure. In this case, the hypothesis is that material loss only occurs after the complete filling of the cavity, but in reality, some of the material is being squeezed out of the mould due to the clearance between the stamp and the mould walls.

The void content of the preform was measured as between 5.8 and 13.4 %, with an average at 8.58% (last line in Table 1, SD = 1.8%). After compression, the porosity content decreased considerably, reaching values between 1.06 and 5.45% (Table 1). Higher pressures provided lower void contents, at the expense of a higher mass of the burr. The goal was to find an optimal applied pressure, where the specimen void content was low, with a reasonable low mass of the burr.

A decrease in the porosity value is observed as the pressure increases. However, the decrease in porosity stagnates and the mass of the burr increases, proportionally, after applying the value of compaction pressure, over 6 MPa. Following this finding, the optimal compaction temperature was yet to be determined. Since most of the voids in the part were present in the edges and corners of the specimen, the experiment was continued at a lower temperature. The hypothesis was that with a lower temperature, the increase in viscosity of the matrix would make the shaping of the specimen more stable. Two more samples were tested at 200 °C, and the results (given in Appendix D) confirmed this hypothesis with a decrease in porosity to 0.26% and 0.61%, respectively. The post-processing window for the 3D-printed CFRP straps was therefore determined to be: 6 MPa compaction pressure, 200 °C compaction temperature for a duration of 10 min (dwell time).

### 3.2. Material Characterisation

In order to characterize the material properties, tensile, flexural, and ILSS tests were performed on the produced coupons according to the standards given in Section 2.3.2, with five specimens per test. Results can be seen in Table 2.

The obtained results exceed the values given in the datasheet [15], except for ILSS. Specimen no. 3 in ILSS experienced an unacceptable plastic failure of the matrix, and was therefore not included in the analysis. Dissimilarities are possible due to different testing methods and a slight change in the material structure and matrix-to-fibre ratio. In particular, our developed post-processing method leads to specimens with a slightly higher FVF (see Table 1), compared to the FVF of 60% given in the datasheet [15]. Some variability might even be due to the different porosity content. The second plate from which flexural and ILSS specimens were cut was additionally tested for its void content. This was, however, not possible with plate no. 1, from which tensile specimens were cut, since the failure during the tensile test was abrupt and the failed specimens were not in a condition to be additionally tested for porosity. The local porosity content value for the second plate was 0.07% with 62.50 % of FVF.

### 3.3. Tensile Experiments on CFRP Straps

**Figure 8:**
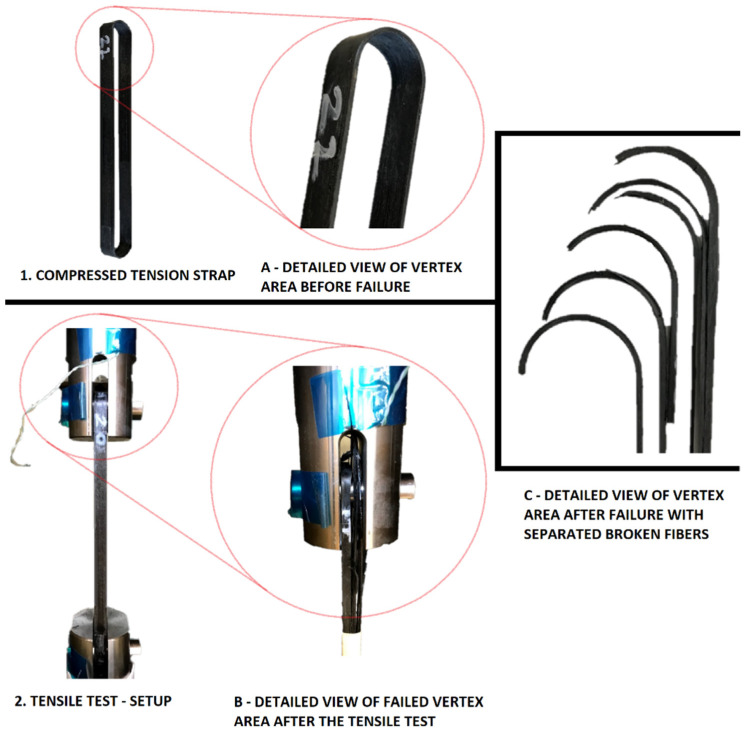
Tensile test setup with compressed strap and a magnified view of the vertex area before and after the failure.

The tensile test setup is presented in Figure 8. Results on the compressed and uncompressed tension straps can be seen in the following tables (Table 3 and Table 4).

The uncompressed tension straps failed prematurely at low load levels, due to the rather bad compaction and adhesion between the layers, low interlaminar strength, and high void content. As explained in Section 2.3.4, a strain gauge was not installed during the tensile test, and the elastic modulus of an uncompressed 3D-printed strap preform could not be determined.

**Figure 9:**
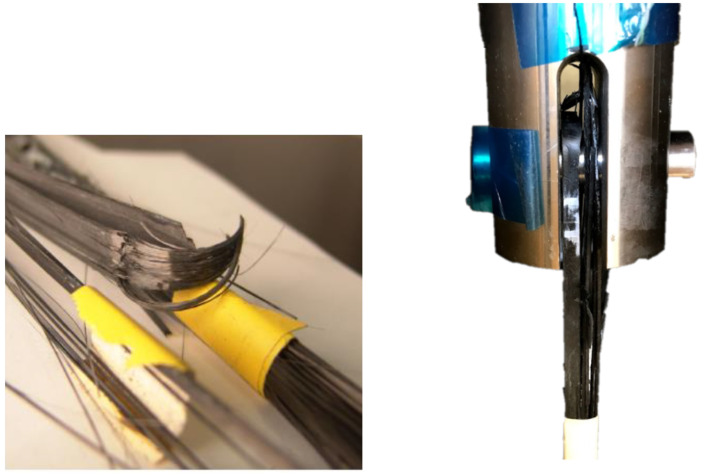
Typical tensile failures of CFRP straps starting at the onset of curvature (vertex).

The values of tensile strength in the case of the compressed straps were between 1132 and 1470 MPa, with an average of 1314.37 MPa. The average tensile strength is 39.2% lower compared to that of the flat coupon specimens (Table 2), and 27% lower than the tensile strength of the material reported by 9T Labs [15]. The observed reduction compared to flat coupons is due to the curved geometry of the strap, since stress concentrations occur at the vertex area of the strap, thus reducing its tensile load-carrying capacity compared to a flat UD coupon [10]. The elastic elongation of the strap that occurs during the tensile loading causes the curved area of the strap to shift along the pin and yet retain its curvature. This phenomenon induces a bending moment in the vertex area, which causes stress concentrations leading to failure at the onset of the strap curvature (Figure 8C and Figure 9). This reduces the mechanical performance of the tension strap compared to the coupon properties [6]. The average value of elastic modulus $E_{∥}$ of the strap-shaft is 132.3 GPa, which agrees quite well with the material datasheet value [15] and the estimated value of 139.1 GPa from the rule of mixtures [10].

**Figure 10:**
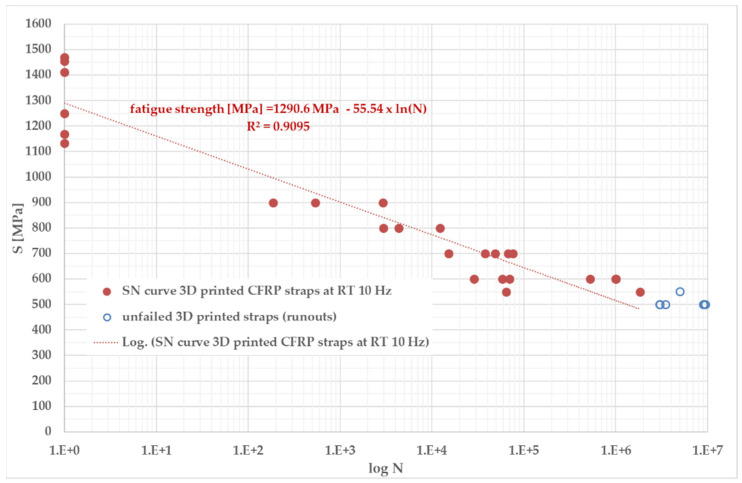
S–N curve for Ti64 pin-loaded CFRP straps, showing the upper stress level S in function of endured load cycles N in logarithmic scale.

Twenty-eight tension straps were finally tested in tensile fatigue. The results can be seen in Appendix A and Figure 10. The fatigue endurance limit of the 3D-printed and then stretched and compacted CFRP straps corresponds to 500 MPa at R = 0.1 and 10 Hz when anchored with Ti64 pins of diameter 20 mm.

The average residual tensile strength of the two straps that sustained 3 million load cycles at the upper stress level of 500 MPa is 1221.64 MPa (the residual tensile strength value for strap No. 50, also sustaining 3.5 Mio load cycles was only 946 MPa, and it was not evaluated due to bad vertex impregnation), and 1221.64 MPa corresponds to 93% of the average tensile strength of the pristine straps being 1314.4 MPa (Table 4). For the four straps that sustained 9 million load cycles at upper stress level of 500 MPa, the residual tensile strength is on average 1243.8 MPa (standard deviation = 117.4 MPa) and is 94.6% of the average tensile strength of the pristine straps (Table 4). These results show that the influence of fretting and fatigue damage (i.e., matrix and fibre cracking) when fatiguing at the endurance limit (500 MPa) for 3–9 million load cycles is limited if one considers the relatively low number of run-out specimens and the corresponding standard deviations.

## 4. Elastic Analysis

**Figure 11:**
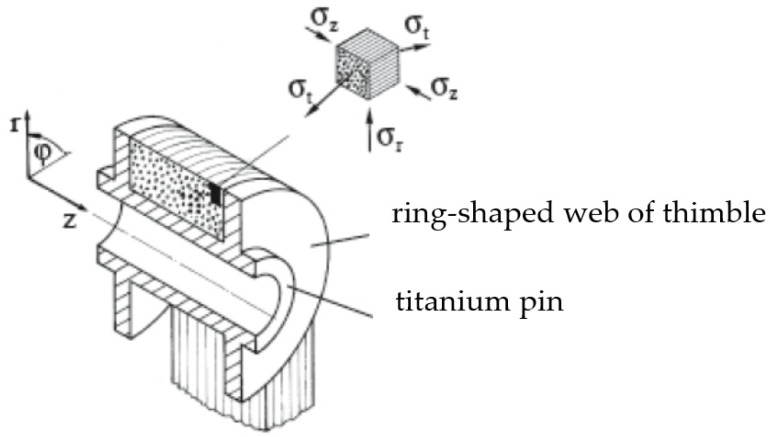
UD CFRP strap supported laterally by ring-shaped webs of the thimbles. Reprinted with permission from [10].

An analytical estimation of the degree of exploitation of the carbon fibres in the 3D-printed/compacted CFRP strap (meant as the ratio of the tensile strength of the strap compared to the average tensile strength of the UD coupons) was performed by an elastic analysis following [10]. This analysis is summarized here, and is based on the model of a thick-walled pipe subjected to internal pressure [34], with the consideration of side supports (ring-shaped webs of the thimbles) made of titanium, as shown in Figure 11.

We are interested in the three-dimensional stress distribution $\left(\sigma_{t}=\sigma_{1},\sigma_{z}=\sigma_{2},\;\sigma_{r}=\sigma_{3}\right)$ in the looped area of the strap supported by the pin and sideways by the ring-shaped webs of the thimble. The curved area of the ‘thick’ CFRP strap is modelled as a cylindrical pipe with carbon fibres in the hoop direction subjected to an internal pressure $p_{i}$. This analysis is performed following an elasto-statics approach ([10], pp. 485–496), which leads to a closed-form solution for the above stress components in the curved part of the unidirectionally reinforced strap. The following assumptions are made in this strap model: The stresses over the width of the strap (parallel to pin’s axis z) are evenly distributed (i.e., constant). Due to the rotation symmetry, stress differences in the strap cannot arise in the hoop direction (t), but only radially (r). Finally, friction of the strap over the thimble and pin is neglected.

The solution for the radial stress—that varies over the radius coordinate—at the apex (top) of the strap is given by:

(3) $$\sigma_{r}\left(r\right)=\frac{-p_{i}\cdot r_{i}^{1+E_{v}}}{r_{i}^{2E_{v}}-r_{a}^{2E_{v}}}\cdot r^{E_{v}-1}+\frac{-p_{i}\cdot r_{i}^{1-E_{v}}}{r_{i}^{-2E_{v}}-r_{a}^{-2E_{v}}}\cdot r^{-E_{v}-1}$$

The maximum radial stress is located at the inner radius $r_{i}$ and corresponds to the haunch pressure $\sigma_{r}\left(r_{i}\right)=-p_{i}$. Knowing the solution (3) for the radial stress distribution $\sigma_{r}\left(r\right)$, the tangential (hoop) stress distribution can be determined via equilibrium on the infinitesimal strap element to:

(4) $$\sigma_{t}\left(r\right)=-p_{i}\cdot E_{v}\cdot \left(\frac{r_{i}^{1+E_{v}}}{r_{i}^{2E_{v}}-r_{a}^{2E_{v}}}\cdot r^{E_{v}-1}-\frac{r_{i}^{1-E_{v}}}{r_{i}^{-2E_{v}}-r_{a}^{-2E_{v}}}\cdot r^{-E_{v}-1}\right)$$

This stress component corresponds to $\sigma_{∥}$ (average normal stress in fibre direction) in the UD ply and is therefore decisive for the strength analysis of the pin-loaded strap and considers the stiffness ratio $E_{v}$:

(5) $$E_{v}=\sqrt{\frac{{\tilde{E}}_{∥}}{{\tilde{E}}_{⊥}}}$$

This corresponds to the square root of the longitudinal to the transverse stiffness coefficients of the unidirectional CFRP ply in its local coordinate system, which is defined by the fibre direction (∥-axis) and the fibre perpendicular axis of the UD ply (⊥).

(6) $${\tilde{E}}_{∥}=\frac{E_{∥}}{\left(1-\nu_{⊥∥}\cdot \nu_{∥⊥}\right)}\;\;\;\;\;{\tilde{E}}_{⊥}=\frac{E_{⊥}}{\left(1-\nu_{⊥⊥}\cdot \nu_{⊥⊥}\right)}$$

The above-cited haunch pressure $p_{i}$ caused by the total force (tension) F loading the pin is given per equilibrium by:

(7) $$p_{i}=\frac{F}{2r_{i}\cdot b}$$

When supporting sideways with the ring-shaped webs of the titanium thimble (or in the test setup shown in Figure 8B with the side surfaces of the loading adapters), we obtain a three-dimensional stress state. The maximum normal (compressive) stress in the axial direction $\sigma_{z}$ appears at the inner radius of the strap. It is computed after [10]—out of $\sigma_{r}$ and $\sigma_{t}$ at the radial coordinate $r_{i}$:

(8) $$\sigma_{z}\left(r_{i}\right)=\left(\upsilon_{⊥⊥}\cdot \frac{\sigma_{r}\left(r_{i}\right)}{E_{⊥}}+\upsilon_{⊥∥}\cdot \frac{\sigma_{t}\left(r_{i}\right)}{E_{∥}}\right)\cdot E_{⊥}$$

The main result of this elasto-static stress analysis is that at the strap inner radius $r=r_{i}$ (Figure 11 shows the coordinate system), we obtain pronounced stress peaks. We now adopt the as called “refined” Puck fibre failure criteria for the unidirectional ply [35], describing the tensile failure of the CFRP strap due to fibre tensile fracture:

(9) $$f_{E}\left(Fb\right)=\left|\frac{1}{R_{∥}^{\pm }}\left[\sigma_{1}-\left(\upsilon_{⊥∥}-\upsilon_{f⊥∥}\frac{E_{∥}}{E_{f∥}}m_{\sigma f}\right)\left(\sigma_{2}-\sigma_{3}\right)\right]\right|=1$$

With $f_{E}\left(Fb\right)$ being the stress exposure value for fibre failure (i.e., $f_{E}\left(Fb\right)=1$ corresponds to fibre fracture in tension or compression) and $R_{∥}^{+}$ corresponding to the tensile strength of the UD coupon ($R_{∥}^{-}$ would be its compressive strength, not relevant for the pin-loaded strap under tension).

An iterative solution of Equation (9) for fibre tensile failure, i.e., $f_{E}\left(Fb\right)=1$ at $r=r_{i}$ for the vertex of the 3D-printed and compacted straps investigated leads to a theoretical strap tensile failure load of F = 52.3 kN $\left(\sigma_{t}=\sigma_{1}=2098\;\mathrm{MPa},\;\sigma_{z}=\sigma_{2}=-63\;\mathrm{MPa},\;\sigma_{r}=\sigma_{3}=-243\;\mathrm{MPa}\right)$. The CFRP strap’s geometric and material properties considered are given in Table 5.

The tensile experiments at room temperature gave an average tensile strength of the CFRP straps of 1314 MPa (Table 4), which is only 70.7% of the above-estimated theoretical strap tensile strength of 1857 MPa (corresponding to F = 52.3 kN).

Reasons for this theoretical overestimation are to be found in the assumptions and idealisations made in Schürmann’s analysis: In [10], an ideal UD ply with perfect UD fibre alignment and fibre–matrix composite action is assumed while the straps investigated show fibre waviness, see Appendix B, and zones with low impregnation of the carbon fibres particularly at the critical curvatures of the straps, Appendix C. In addition, the presence of friction between the pin and the UD strap ply in the experiments leads to stress concentrations along the curvature [36]. One also needs to consider that the tensile strength $R_{∥}^{+}$ of the CFPA12 coupons is probably higher than the strength of the shaft of the strap (due to better compaction in the thickness direction and fibre alignment in the coupon) and that Schürmann’s model does not consider residual stresses due to differential thermal fibre/matrix expansions in the FFF process nor local bending effects at the strap’s vertex: As discussed in Section 3.3, the tensile load transfer at the strap end curvatures leads to local bending stresses in the vertex area that are superimposed to the above $\sigma_{t}=\sigma_{1}$_-_stress peak at $r=r_{i}$ [10]. This has been analysed via finite element modelling in [6] for geometrically very similar CFRP straps made of IMS60 carbon fibres with an epoxy matrix. This work showed a local $\sigma_{1}$ increase of 30% leading to premature strap failure at the onset of its curvature (the as-called “strap vertex”, see Figure 9).

## 5. Discussion and Conclusions

The results presented in this study show a good potential for further research on 3D-printed CFRP straps under the assumption of an appropriate compaction procedure. In standard tensile tests at room temperature, the average strap strength was 1314 MPa (standard deviation of six specimens 149 MPa). This corresponds to 70.7% of the theoretical tensile strength of the strap analysed with Schürmann’s elasto-static analysis [10] considering refined Puck fibre fracture criteria [35]. The deviation is explained by model assumptions and UD ply imperfections.

Without a post-processing step consisting of a longitudinal stretching followed by a transverse consolidation, the average strap strength was only 264 MPa (standard deviation of five specimens 56 MPa). This is only 14.2% of the theoretical value according to Schürmann and 20% of the tensile strength of a consolidated strap. This highlights the need for appropriate post processing.

The SN curve obtained in the tensile fatigue of 28 post-processed strap specimens at a loading frequency of 10 Hz (R = 0.1) and using Ti64 pins to anchor the straps is similar to that obtained for OAA laminated straps based on unidirectional carbon fibre epoxy prepregs and investigated previously by the corresponding author’s Empa laboratory [6]. A fatigue endurance limit for the thermoplastic matrix straps of 500 MPa could be determined. Two respective four samples loaded with a maximum tensile stress of 500 MPa were able to withstand $3\times 10^{6}$ and 9$\times 10^{6}$ fatigue load cycles, and their remnant tensile strengths (1222 MPa after 3 million load cycles and 1244 MPa after 9 million load cycles) were only slightly lower than that of pristine straps. The fatigue endurance limit of 500 MPa at R = 0.1 and 10 Hz would correspond to approximately 38% of the CFRP straps’ ultimate tensile strength.

As a comparison, straps made with a stronger IMS60 carbon fibre that were conventionally produced with an out-of-autoclave process (epoxy matrix) have a fatigue endurance limit of 750 MPa, which corresponds to 46% of their ultimate tensile strength [6].

The main drawback of the processing method presented in this paper is the necessary post processing after 3D printing (FFF) of the CFRP straps. This includes an axial stretching followed by a transversal post compaction (i.e., a 6 MPa compression of the strap at 200 °C in the width direction) in a complex and expensive steel mould. This tool needs to be designed and produced for each strap geometry in a practical application, e.g., for network arch bridge straps with a diameter of 33 mm and lengths of several metres [4]. This rather demanding stretching and compaction, which is necessary to exploit the carbon fibre strength in the looped tensile element, is a clear limitation of the 3D printing technique by FFF of thermoplastic matrix straps as it makes the strap production expensive and inflexible. It therefore greatly compromises the advantages (geometric freedom, fast production) of printing CFRP laminates with a thermoplastic matrix. Novel research from Japan [37] is trying to avoid this additional consolidation post-processing step by integrating compaction in the 3D printing head with an advanced additive manufacturing device.

The presented results on the tensile fatigue performance of 3D-printed, axially stretched, and transversally compressed CFRP straps look promising, and further research will focus on the improvement of the manufacturing process of the strap and mould design in order to improve fibre impregnation (Appendix C) and to reduce fibre waviness (Appendix B). With further optimization of the post-compacting mould design, there is potential to further enhance the UD strap’s quality and therefore the tensile and fatigue performance of the CFRP strap.

For a better exploitation of the geometrical flexibility of the presented FFF 3D printing process, further developments focussing on the topological optimization of the curved end areas of tension straps should be performed in the future with the aim to reduce the discussed stress concentrations at the strap vertex.

## Figures and Tables

**Table 1:** Compression test results of the first five specimens, compressed at 210 °C and 10 min of dwell time, with various compaction pressures. Appendix D gives the complete results of the experiments including measured standard deviation at each pressure level.

Sample	Compaction Press. (MPa)	Temp. (°C)	Compress. Ratio (%)	Loss of Material (%)	Density of Comp. Part (g/cm^3^)	Fibre Vol. Fraction (%)	Porosity Vol. Fraction (%)
1	10	210	41.09	15.63	1.51	60.99	1.16
2	9	210	38.29	10.07	1.50	61.41	1.06
3	6	210	34.83	5.43	1.49	61.57	1.29
4	4	210	29.85	4.02	1.48	60.43	4.12
5	2	210	29.03	3.21	1.48	59.56	5.45
Preform	-	-	-	-	-	43.41	8.58

**Table 2:** Material characterisation results.

Sample	Flexural Modulus (GPa)	Flexural Strength (MPa)	Tensile Modulus (GPa)	Tensile Strength (MPa)	In-Plane Shear Strength (MPa)
1	117	1050	139.10	2049.26	41.5
2	117	972	146.99	2163.42	41.5
3	118	949	150.55	2391.23	-
4	117	1010	147.85	2123.93	42.3
5	117	1120	135.25	2087.55	41.2
Average	117.20	1020.20	143.95	2163.08	41.63
St. deviation (SD)	0.45	67.72	6.47	134.39	0.47
Datasheet [15]	112 ± 2	768 ± 28	133 ± 3	1820 ± 12	45
Standard	[32]		[31]		[33]

**Table 3:** Results of the tensile tests of the uncompressed 3D-printed CFRP preforms under quasi-static conditions.

Tension Strap	Height (mm)	Thickness (mm)	Cross-Section Area (mm^2^)	$F_{max}$ (N)	$\sigma_{t}$ (MPa)
1	11.85	1.25	29.63	8563	289.00
2	11.87	1.24	29.44	6002	203.87
3	12.00	1.22	29.28	9796	334.56
4	11.95	1.22	29.16	8196	281.07
5	11.77	1.29	30.37	6401	210.77
Average	11.89	1.24	29.58	7791.60	263.85
St. deviation	0.09	0.03	0.48	1574.22	55.55

**Table 4:** Results of the tensile tests of the stretched and compressed 3D-printed CFRP straps under quasi-static conditions. The six strap specimens are depicted in Appendix C.

Tension Strap	Width (mm)	Thickness (mm)	Cross-Section Area (mm^2^)	$F_{max}$ (N)	$\sigma_{t}$ (MPa)	*E* (GPa)
CFRP-18	11.32	1.33	30.11	42,477	1410.67	-
CFRP-19	10.98	1.30	28.55	41,523	1454.50	-
CFRP-20	10.67	1.31	27.96	34,942	1249.92	131.88
CFRP-21	10.61	1.31	27.80	32,499	1169.10	135.75
CFRP-22	9.90	1.31	25.94	38,129	1470.01	130.95
CFRP-23	11.01	1.30	28.63	32,306	1132.04	130.54
Average	10.75	1.31	28.16	36,979.33	1314.37	132.28
St. deviation	0.49	0.01	1.36	4432.95	149.42	2.38

**Table 5:** CFRP strap’s geometric and material properties considered in the elastic strength prediction after [10,36]. Sources for the data assumed are given in the last row of the table.

$E_{∥}$ (MPa)	$E_{f∥}$ (MPa)	$E_{⊥}$ (MPa)	$\nu_{⊥∥}$ (−)	$\nu_{∥⊥}$ (−)	$\nu_{⊥⊥}$ (−)	$\nu_{f⊥∥}$ (−)	$R_{∥}^{+}$ (MPa)	$m_{\sigma f}$ (−)	$r_{i}$ (mm)	$r_{a}$ (mm)	b (mm)
144,000	231,000	9650	0.28	0.015	0.042	0.1	2163	1.1	10	11.31	10.75
[10,15]	[27]	[10,27,28]	[10,27]	[10]	[10]	[10]	Table 2	[10]	Table 4		

**Figure A1:**
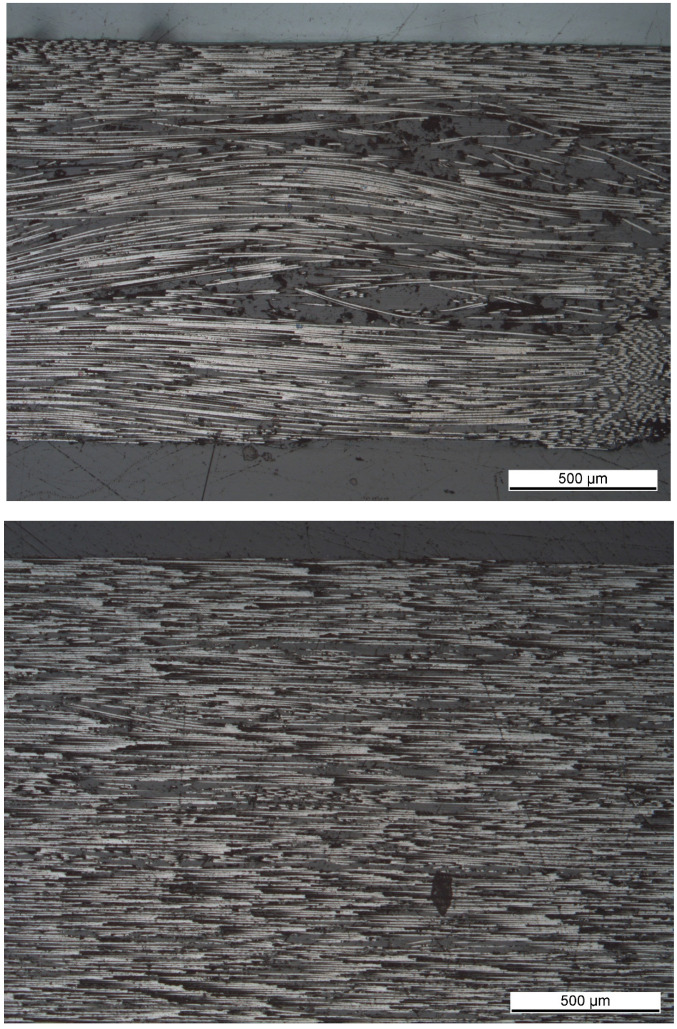
Local carbon fibre waviness for 3D-pinted and post-compacted straps along strap length.

**Figure A2:**
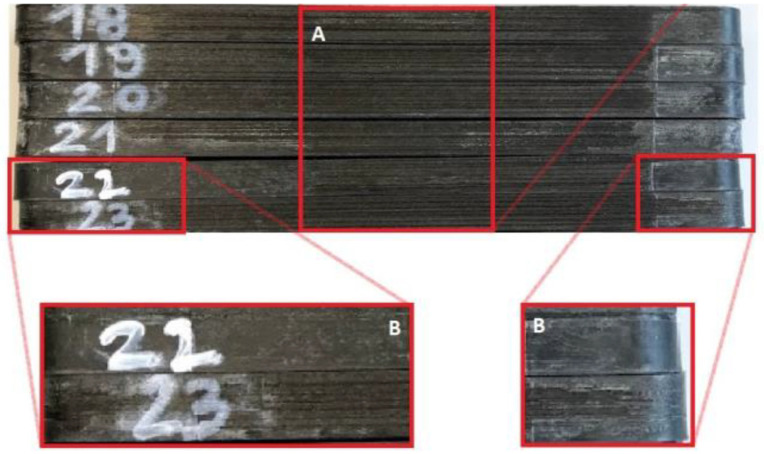
Tensile test specimens of 3D-printed CFRP straps before testing, details show curvature areas with partially poor impregnation.

## Data Availability

The majority of the data can be found in the MSc thesis of T. Vidrih submitted in 2021 to the Dept. of Mechanical Engineering of the University of Ljubljana.

## Appendix A

Supplemental data regarding the main results of the tensile fatigue tests of Ti64 pin-loaded 3D-printed CFRP straps are shown in Table A1, Table A2, Table A3 and Table A4.

**Table A1:** Results of the tensile fatigue tests of compressed 3D-printed tension straps.

Strap	$F_{u}$ (kN)	$\sigma_{u}$ (MPa)	$\mathrm{Cycle}\;N_{f}\;$(-)	$\mathrm{Load}\;\mathrm{Ratio}\;R=\sigma_{u}/\sigma_{l}\;$(-)	Frequency f (Hz)	Failure
CFRP-24	20.04	700	15,238	0.1	10	yes
CFRP-25	14.10	500	9,020,000	0.1	10	no
CFRP-26	17.44	600	58,074	0.1	10	yes
CFRP-27	15.69	550	63,994	0.1	10	yes
CFRP-28	14.61	500	3,010,000	0.1	10	no
CFRP-29	17.16	600	70,000	0.1	10	yes
CFRP-30	17.35	600	995,075	0.1	10	yes
CFRP-31	14.46	500	3,010,000	0.1	10	no
CFRP-32	17.42	600	525,836	0.1	10	yes
CFRP-33	17.31	600	1,001,625	0.1	10	yes
CFRP-34	19.76	700	76,292	0.1	10	yes
CFRP-35	20.08	700	37,865	0.1	10	yes
CFRP-36	20.32	700	48,529	0.1	10	yes
CFRP-37	20.27	700	67,482	0.1	10	yes
CFRP-38	14.74	500	3,010,000	0.1	10	no
CFRP-39	14.02	500	9,050,000	0.1	10	no
CFRP-40	14.46	500	9,100,000	0.1	10	no
CFRP-41	14.24	500	9,453,050	0.1	10	no
CFRP-42	16.25	550	1,841,764	0.1	10	yes
CFRP-43	15.80	550	5,000,000	0.1	10	no
CFRP-44	24.81	800	4350	0.1	10	yes
CFRP-45	23.71	800	2957	0.1	10	yes
CFRP-46	23.30	800	12,240	0.1	10	yes
CFRP-47	25.19	900	186	0.1	10	yes
CFRP-48	25.16	900	535	0.1	10	yes
CFRP-49	23.13	900	2918	0.1	10	yes
CFRP-50	13.82	500	3,481,000	0.1	10	no
CFRP-51	16.83	600	28,674	0.1	10	yes

## Appendix B

Optical microscopy figure taken along a CFPA strap showing carbon fibre waviness despite post processing by axial stretching.

## Appendix C

Photographs showing dry surface spots (denominated with “B) with incomplete impregnation of the AS4 carbon fibres by PA12 in the critical end zones and curvatures of the straps.

## Appendix D

Supplemental data for a compaction temperature of 210 °C giving the complete results of the fibre and porosity volume fraction analysis, which are summarized as average values in Table 1 of Section 3.1.

**Table A2:** Compression test FVF results of the first five specimens, compressed at 210 °C and 10 min of dwell time, with various compaction pressures (sample 1 with 10 MPa, 2 with 8 MPa, 3 with 6 MPa, 4 with 4 MPA, 5 with 2 MPa).

	Fibre Volume Fraction (FVF) (%)—Data						
Sample	1	2	3	4	5	St. Deviation (%)	Average (%)
1	59.44	63.32	61.71	60.5	60	1.55	60.99
2	59.17	61	64.08	60.38	62.42	1.90	61.41
3	61.13	61.62	61.04	61.79	62.29	0.51	61.57
4	60.28	61.72	58.17	61.49	60.5	1.41	60.43
5	60.53	59.36	60.58	58.89	58.44	0.96	59.56

**Table A3:** Compression test porosity results of the first five specimens, compressed at 210 °C and 10 min of dwell time, with various compaction pressures (sample 1 with 10 MPa, 2 with 8 MPa, 3 with 6 MPa, 4 with 4 MPA, 5 with 2 MPa).

	Porosity Volume Fraction (%)—Data						
Sample	1	2	3	4	5	St. Deviation (%)	Average (%)
1	0.49	0.56	4.21	0.33	0.21	1.71	1.16
2	0.74	1.06	0.4	0.75	2.34	0.75	1.06
3	0.53	0.55	0.39	0.37	4.6	1.85	1.29
4	3.85	5.78	4.01	4.56	2.38	1.23	4.12
5	3.76	6.79	8.46	6.41	1.84	2.63	5.45

Results of the two additional samples tested at 200 °C:

**Table A4:** Compression test porosity results of the second two specimens, compressed at 200 °C and 10 min of dwell time, with various compaction pressures (sample 1 with 10 MPa, 2 with 8 MPa, 3 with 6 MPa, 4 with 4 MPA, 5 with 2 MPa).

	Porosity Volume Fraction (%)—Data						
Sample	1	2	3	4	5	St. Deviation (%)	Average (%)
1	0.22	0.16	0.26	0.16	0.49	0.14	0.26
2	0.86	0.53	0.48	0.36	0.83	0.22	0.61

**Table A5:** Compression test FVF results of the second two specimens, compressed at 200 °C and 10 min of dwell time, with various compaction pressures (sample 1 with 10 MPa, 2 with 8 MPa, 3 with 6 MPa, 4 with 4 MPA, 5 with 2 MPa).

	Fibre Volume Fraction (%)—Data						
Sample	1	2	3	4	5	St. Deviation (%)	Average (%)
1	61.51	62.05	63.29	63.91	64.46	1.24	63.04
2	63.79	64.95	64.07	64.34	61.92	1.14	63.81
